# Coping strategies of students for anxiety during the COVID-19 pandemic in China: a cross-sectional study

**DOI:** 10.12688/f1000research.25557.1

**Published:** 2020-09-10

**Authors:** Mohammad Nurunnabi, Syed Far Abid Hossain Hossain, Karuthan Chinna, Sheela Sundarasen, Heba Bakr Khoshaim, Kamilah Kamaludin, Gul Mohammad Baloch, Areej Sukayt, Xu Shan

**Affiliations:** 1 Prince Sultan University, Riyadh, 11586, Saudi Arabia; 2 College of Business Administration, International University of Business Agriculture and Technology, Dhaka, Bangladesh; 3 Faculty of Health and Medical Sciences, School of Medicine, Taylor's University, Subang Jaya, Malaysia; 4 Xi'an Jiaotong University, Shaanxi, China

**Keywords:** Coping strategy, Students, University, China, COVID-19, Corona virus diseases

## Abstract

**Background:** COVID-19 has severely affected university students everywhere in the world. Due to fear of infection, government and local authorities in China immediately closed academic institutions and tried to find survival techniques to cope with market turbulence. COVID-19 was present in China at the end of 2019. However, little attention has been paid by researchers to coping strategies during the COVID-19 pandemic, and few measures were taken to assess the coping strategies of university students, specifically following the closure of their institutions. To address this gap, this study attempted to discover the coping strategies of Chinese students during the COVID-19 pandemic in China.

**Methods:** We conducted an online survey using a semi-structured questionnaire with a simple random sampling technique and received 559 responses. The survey questions captured information about students’ lives during the COVID-19 outbreak, actions to control anxiety, and what students care about during the pandemic. The associations between coping strategies used and levels of anxiety were tested using analysis of variance (ANOVA) procedures. SPSS Statistics v27 was used for statistical analysis in this study.

**Results:** The university students reported that coping strategies and survival techniques were required due to high levels of anxiety and psychological pressure during the COVID-19 pandemic. Most of the respondents reported the prompt closure of their academic institutions due to COVID-19. Psychological concerns, such as lack of sleep, emotional support, mental support and social appeal, were also reported.

**Conclusions:** This is one of the very first studies on coping strategies for anxiety in China. The study reveals that university students employ a number of coping strategies in relation to COVID-19, but also suggests a need to strengthen such strategies in this population. However, the study was limited to a small number of provinces in China, which may affect the generalizability of the research.

## Introduction

**Figure 1.**
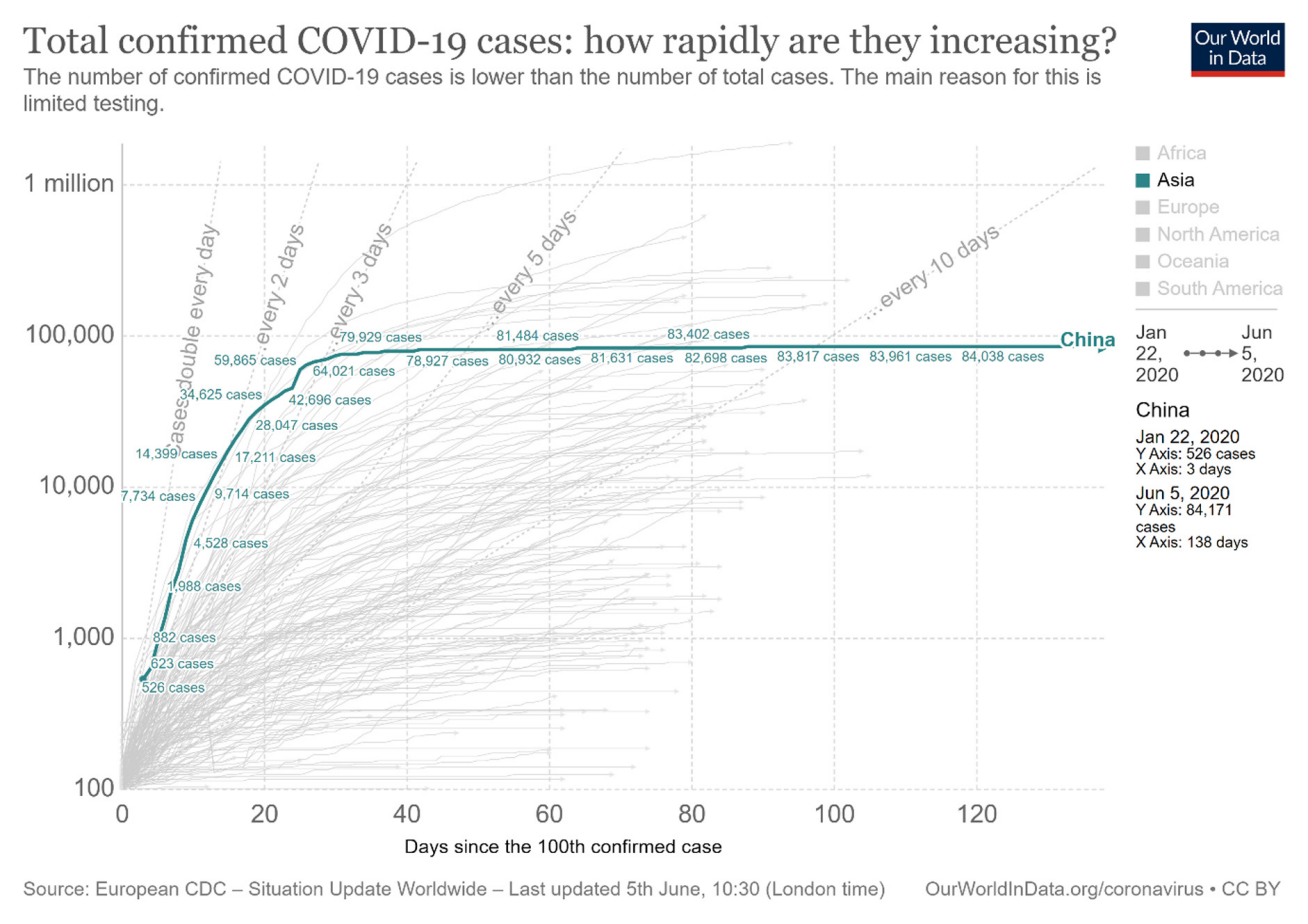
Increasing trend of COVID-19 in China ^2^. This chart has been reproduced under the terms of the Creative Commons Attribution 4.0 International license (CC-BY 4.0).

The COVID-19 outbreak infected mainland China and the surrounding regions drastically at the end of 2019. To cope with the infection, the Chinese government executed severe control measures, including an expansion of the national holiday, immediate lockdown and strict post-lockdown measures as part of an effort to prevent a second coronavirus wave. In addition, the mobility of the population was controlled with severe restrictions at a national level, and social distancing was monitored as well as encouraged with a compulsory quarantine period of two weeks for returning migratory personnel. Reflecting these containment measures, the economy contracted by 6.8% in the first quarter (CNBC 2020 ^1^). The increasing number of cases of COVID-19 in China between January and June 2020 can be seen in Figure 1.

In the middle of February, the Chinese government gradually decided to remove restrictions on mobility of the population and other activities, prioritizing some provinces and various groups of people based on risk calculations. Most trade and universities reopened countrywide with careful observation during the stages of reopening. However, social distancing rules remained in place as did the mandatory use of a facial mask. Entering the country from overseas also remained restricted in some situations to control the importation of cases. The danger of new infections from imported cases has been highlighted by the local community and the government has taken the matter seriously and restricted foreign entry to China ^1^. Apart from that, regular temperature checking and personal health QR codes were imposed on a compulsory basis for traveling locally and entering public places in order to cope with the pandemic (WHO 2020 ^2^).

**Figure 2.**
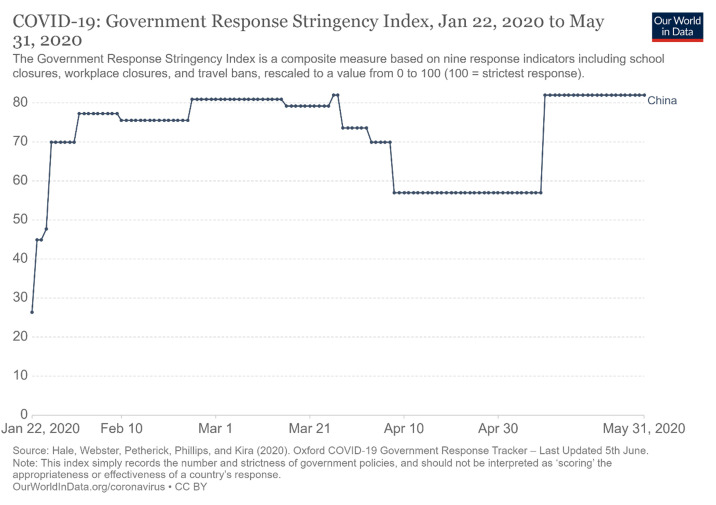
Government response stringency index ^2^. This chart has been reproduced under the terms of the Creative Commons Attribution 4.0 International license (CC-BY 4.0).

Figure 2 shows the Chinese government’s response stringency index. The government response included the closure of universities and other academic institutions, factories and offices, and travel restrictions. The responses are measured on a scale of 10 to 100, and the graph in Figure 2 shows a very high rate of restrictions from March to May, 2020.

**Figure 3.**
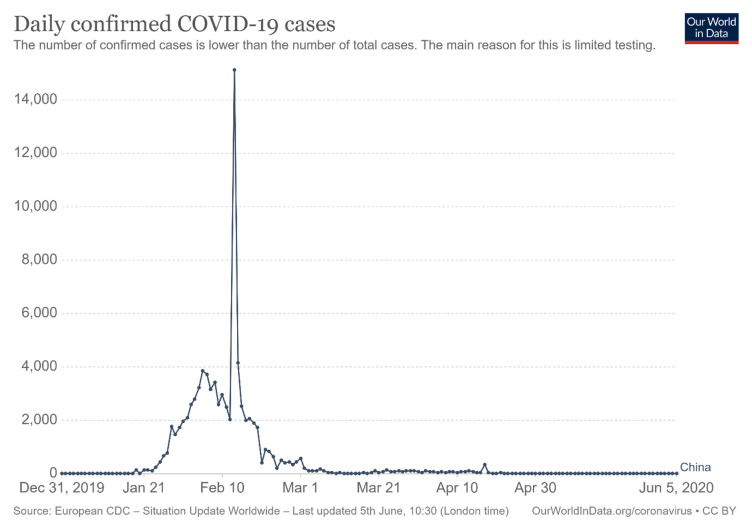
Daily confirmed COVID-19 cases ^2^. This chart has been reproduced under the terms of the Creative Commons Attribution 4.0 International license (CC-BY 4.0).

Figure 3 shows the daily confirmed cases of COVID-19 in China; February had the highest number of confirmed cases. Even though the daily confirmed cases has decreased recently, strategies for coping with the pandemic are still under the control of the Chinese government. For example, no international students have been allowed to return to university. If a student returned, they would receive serious penalties, as announced by some universities ^3^. As a result, students are struggling to complete their studies on time, and many students are affected. Even if students are not infected with the virus, they may be depressed as their studies have been disrupted and they cannot finish their studies on time. Online teaching and graduation opportunities have been provided by some universities, but many students are not capable of finishing their studies in this way. Therefore, this study aimed to shed light on the coping strategies used by university students in China during the COVID-19 outbreak. A coping strategy in this article is defined as the behaviors, thoughts, and emotions that students use to adjust to the changes that occur in their life, e.g. writing issues down in order to process them during COVID-19.

## Literature review

### Coping of Chinese citizens in a historical context

Chinese people have been familiar with various pandemics over the years. However, COVID-19 is the deadliest pandemic in China’s recorded history. In 2003, Severe Acute Respiratory Syndrome (SARS) affected China, especially in Beijing ^4^. Although SARS was not as dangerous as COVID-19, it was considered as a “warning” ^5^ to society, and the infection was controlled well within the country.

Mental health has been severely affected by the COVID-19 infection owing to fear of the pandemic, and various coping strategies are observed ^6^, affecting mental health care, human care, psychological crisis control measures, and intervention in COVID-19 ^7^. Previous research surrounding coping strategies has focused mainly on SARS; however, as COVID-19 is novel, the socio-psychological impacts of COVID-19 are still under investigation. Coping strategies are not only urgently required, but also need to be adapted to university students requiring an emergency strategy to be decided on by policy makers ^8^.

Coping strategies for COVID-19 cannot be short term. There may be a long time frame, since subsequent waves of COVID may arise. While researchers are continuously working to provide the right medicine, vaccinations and recovery measures, little research has been conducted on the coping strategies of students studying in China. SARS killed hundreds of people in China about ten years ago, and people struggled to cope with the situation ^9^; the same situation arose in Hong Kong with Middle East respiratory syndrome (MERS) ^9^. Anxiety among college students in Hong Kong was investigated by previous researchers ^10^ during the SARS outbreak; however, the coping strategies of Chinese students currently studying in mainland China are still elusive.

### Coping strategies and mental health issues in China during COVID-19

One of the key aspects associated with coping strategies during COVID-19 in China is mental health. Different age groups are observed to face different mental challenges in coping with the pandemic. The way in which the unpredictable effects of COVID-19 were dealt with in China provide a lesson in how to cope with pandemic situations calmly. Among the general population in China, immediate action was taken by the local and central authority. This is known as an immediate psychological response, and can help people to cope with the pandemic situation ^11^. However, there is little research into socio-psychological effects and the relevant coping strategies. So far, personal reactions have been examined in the context of community action taken to tackle the Wuhan COVID-19 outbreak ^7^. Coping strategies are hard to implement anywhere; however, in China, the people supported and responded well to measures to cope with the pandemic during COVID-19 ^11^. The fact is, if a person’s mental health can be supported, coping strategies for fighting against COVID19 will be comparatively flexible as the COVID-19 outbreak is treated as a psychological problem. In order to implement coping strategies, psychiatrists have played an important role in society. The role of telehealth services has been revealed as an ideal coping strategy recently ^12^. The coping strategies are ongoing, and based on industry and situation; as a result, the socio-psychological impact of the coping strategies on students studying in China should be investigated.

### Coping strategies in Chinese educational settings

Chinese educational institutions played an exemplary role in controlling local as well as international students. Coping strategies depend on the mental health of students, which has varied a lot during the COVID-19 epidemic. Not only local Chinese students but also international students struggled to cope with the pandemic in order to achieve their desired academic performance. Different coping strategies are observed among both Chinese and international students. Local students mostly returned to their hometown as long as the local transport was operating. On the other hand, university authorities did not decide whether or not international students should go back to their own country; however, the students were instructed to be safe and not to travel anywhere without informing the responsible teacher. Following this decision, students in different cities in China faced mental upset and couldn’t decide what to do, as revealed on various social media ^13^. The students exposed their problems and coping difficulties in specific cities like Wuhan, where the situation was unsettling from the end of January until the end of February. Coping strategies are also negatively associated with psychosocial problems, as stated in recent research ^14^ due to the high risk of infection and anxiety among health workers. Finally, coping strategies in China during the COVID-19 outbreak are utilized differently by two different groups of students. According to a longitudinal study ^15^, one group of students decided to stay in the dormitory and not to move anywhere during the epidemic. This coping strategy was not based on students’ concern only for themselves; rather, this group thought about others such as their parents and other family members. Another group of students assumed that they were safe and they decided to return to their homes. This is another coping strategy observed among students studying in China at the moment.

## Methods

### Survey

Initially, the questionnaire was piloted and validated by seven professors from various countries. There were no changes during the validation stage. An online semi-structured survey was developed by the authors. The survey was administered using a Chinese website called wjx.cn forms, which are similar to Google or Microsoft forms. The questionnaire was designed in English and then translated into Chinese with the help of a Chinese native speaker, who is also expert in English. The authors decided to use the Chinese version as native speakers feel more comfortable answering in Chinese. The authors re-translated the Chinese version using Google Translate to make sure that the original meaning was retained. Four coping strategies were assessed: “Seek social support,” “Avoidance,” “Mental disengagement” and “Humanitarian” were tested. The items were measured on a scale of 1 to 4; 1 = never/rarely, 2 = sometimes, 3 = often and 4 = very often/always. This is similar to the self-rating anxiety scale (SAS) ^16^. The survey is available as *Extended data* ^17^.

The survey was developed by the authors and was in two parts. Part 1 collected socio-demographic variables, including age group, gender, field of study, level of study, current accommodation and current stay (with whom). Part 2 contained the following sections related to anxiety: level of anxiety; coping strategies; and anxiety level with coping strategies. There were 20 multiple choice questions in the personal situation section that were rated on a scale from “Never” to “Always”; or “Not applicable” to “More than ever.”

### Participants

The link to the survey was sent to students in various academic and study groups who are mainly research focused. A simple random sampling technique and various social networking sites (SNSs), including WeChat, were used. Inclusion criteria were that the students had to have access to the internet, were adults (> 18 years old), were able to understand Chinese, and were willing to give informed consent to participate.

On receiving and clicking the survey link, the students accessed information about the survey purpose, which included consent information. After they agreed, using a ‘yes’ button to consent to participating in the survey, they filled in their demographic information, and then the next set of questions appeared. The participants had to answer each section’s questions in order to proceed to the next section.

The participants were stimulated to join the survey with a reward offered (a total of 1,000 Chinese Yuan, or 2 RMB per participant) via red packet 红包 (hóngbāo). The red packet is a system well-known in China for distributing random amounts of money to people on SNSs.

### Data collection

Data collection was initiated from May 26 ^th^ to June 3 ^rd^, 2020. Initially, the survey was sent to students in the Shaanxi province only; however, owing to a slow response, this was expanded to various provinces of China, including Shaanxi, Hubei, Beijing, Heilongjiang and Guangdong.

### Data analysis

The associations between coping strategies used and levels of anxiety were tested using ANOVA procedures. In particular, SPSS v27 was used for statistical analysis in this study.

### Ethical considerations

All work involving human participants was approved by the Prince Sultan University (PSU)’s Institutional Review Board Committee (approval reference: PSU IRB-2020-04-0038).

## Results

We received 559 responses. The demographic details of the participants are represented in Table 1. Among the 559 respondents, 40.4% were women and 50.3% were in the 18–22 age group. In total, 58.5% of respondents were economic management students and more than half were undergraduate students. While 26.8% of respondents stayed in the university dormitory, 17.9% lived in a rented apartment without family members.

**Table 1.** Demographic information of the respondents.

Variable		*n*	%
Gender	Male	226	40.43
	Female	333	59.57
Age	18–22	281	50.27
	23–27	192	34.35
	28–32	66	11.81
	33+	20	3.58
Major	Science and Engineering	124	22.18
	Economic management	327	58.5
	Social Science/Art	58	10.38
	Others	50	8.94
Level of education	Undergraduate	335	59.93
	Graduate	157	28.09
	PhD	51	9.12
	Professional	13	2.33
	Other	3	0.54

### Factors associated with students' anxiety and coping strategies during the epidemic

**Figure 4.**
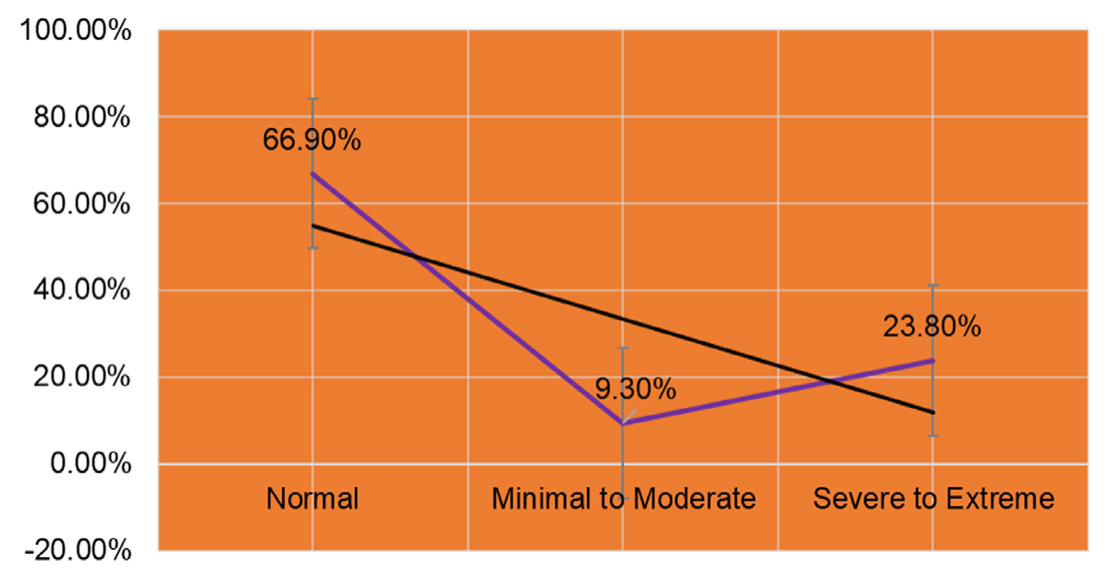
Levels of anxiety reported by students.

In this study, we wanted to know what strategies the students used in coping with anxiety during the COVID-19 pandemic. In total, 66.90% of the students reported experiencing “normal” anxiety, and 23.80% reported it as “severe to extreme” ( Figure 4). Table 2 shows the results showing how much each of the four coping strategies was used by the respondents. Overall, the usage of all four strategies was moderate to low. The distributions were fairly normal (skewness < 2, kurtosis < 7). Overall, the students practiced more mental engagement strategies and fewer social support strategies.

**Table 2.** Coping strategies used by students during the COVID-19 pandemic.

Coping strategy	Mean ± SD	Median	Skewness	Kurtosis
Seek social support	2.28 ± 0.95	2.00	0.356	1.033
Avoidance	2.34 ± 0.92	2.00	0.275	1.019
Mental disengagement	2.55 ± 0.75	2.50	0.361	0.641
Humanitarian	2.33 ± 0.90	2.00	0.391	0.947

In the ANOVA analyses, the variances were similar ( Table 3). All four strategies were significantly associated with levels of anxiety. The usage of all four coping strategies was higher in the “severe to extreme” group compared to the “normal” and “minimal to moderate” groups.

**Table 3.** Anxiety level and coping strategies in students during the COVID-19 pandemic.

Coping strategy	Anxiety Level	Mean ± SD	F	P
Seek social support	Normal Minimal to moderate Severe to extreme	1.90 ± 0.70 ^a, b^ 1.98 ± 0.56a 3.48 ± 0.95b	278.482	<0.001
Avoidance	Normal Minimal to moderate Severe to extreme	1.98 ± 0.73a,b 2.13 ± 0.44 a 3.45 ± 0.59 ^b^	233.283	<0.001
Mental disengagement	Normal Minimal to moderate Severe to extreme	2.27 ± 0.54a,b 2.20 ± 0.45a 3.48 ± 0.55 ^b^	266.619	<0.001
Humanitarian	Normal Minimal to moderate Severe to extreme	1.99 ± 0.66a,b 2.01 ± 0.62 a 3.42 ± 0.90 ^b^	246.697	<0.001

Note: ^a, b^ pairwise differences

## Discussion

The findings of the study indicate that the mental or cognitive health condition of university students in China needs to be further ascertained in order to elucidate the actual psychological impact due to the COVID-19 pandemic.

The outbreak of COVID-19 may be stressful and significantly affect individuals’ mental health. Symptoms of mental health issues, as well as social stressors such as uncertainty due to COVID-19, affect the coping tendency of students. The results of this study reveal that coping strategies are diverse based on demographic and regional difference in China.

The world has occasionally observed numerous life-threatening epidemics, such as COVID-19. It is worth noting that a failure to adopt coping strategies may seriously affect students’ academic achievement due to the unpredictability of COVID-19 ^18^. Financial problems, a lack of health care facilities and safety issues are also major concerns affecting coping strategies in China. Current research ^19^ has conducted a parallel study based on Indian citizens. In that study in India, the authors mentioned a high demand for and shortages of hand sanitizers, hand wash, and facial masks, indicating people’s growing concern to avoid COVID-19 infection. Every epidemic has its unique characteristics in terms of causality, risk and control measures. COVID-19 is not different; however, socio-psychological pressure is acute. Back in 2015, during the Ebola virus outbreak in Ethiopia, coping measures and awareness were less satisfactory than the results of this study found in China.

Coping strategies are directly associated with anxiety levels. The most extreme anxiety level in this study is reported by 20.9% of students, which is more than one-fifth of the total sample size. Students’ ability to seek social support, their isolation and mental disengagement and responsiveness to humanitarian issues are all assessed when measuring their copying strategies. For all the four cases, *p*-value is <0.001 between coping strategies and anxiety levels. This result indicates a strong positive relationship between coping strategies and the anxiety level of students. The contributors in this study had a satisfactory level of awareness concerning COVID-19 and the majority of them took the recommended preventive measures ^20^.

In China, local government and community members emphasized preventive measures such as movement control, temperature checks, wearing respiratory masks, and so on. The study participants reported that most of their school or academic unit were using online teaching during the epidemic. A total of 93.92% reported that their academic institution conducted online lectures, training sessions, video conferencing, competitions and award ceremonies and so on via online links. A total number of 246 participants (44.01%) reported that their academic institutions started online classes in the second month of the outbreak. Due to uncertainty about future study continuation, students reported more anxiety than usual (more than 63%). Similarly, sleeping problems and nightmares were also reported at a higher percentage than before (more than 40%). In this study, 68.87% of students reported unclear learning tasks in the current academic year or semester due to COVID-19. Results from the univariate analysis indicate that female students were more affected than male students in terms of anxiety and stress. However, it is interesting to note that 66.9% of students reported their anxiety level as normal during COVID-19. This is consistent in the sense that knowledge can be recovered from any disruption ^21^ like a pandemic. On the other hand, mental disengagement seems crucial in the analysis. Students from business and management majors have a higher level of anxiety, which contradicts previous studies claiming that music students were more anxious ^22^. Those staying in rented apartments were prone to severe anxiety compared to those staying at home, which indicates that students may feel psychologically worse if they did not stay with their families. The discussion is consistent with the result of the overall study in terms of level of anxiety and coping strategies presented in Table 3.

## Limitations, conclusion and future research

The study has a few limitations that can be addressed in order to further investigate the phenomenon. First, the study is limited to students in China from different universities and majors. Also, the respondents are selected randomly due to online data collection from various provinces that were not affected equally. The respondents are Chinese students mostly from various recognized universities. Thus, findings should not be generalized to the overall student group. Future studies should investigate a proportional study among school, college and university students. In addition, the most affected and least affected areas should be separately investigated to fully analyze the phenomenon. In the future, a mixed methodology approach may further help with in-depth investigation of the phenomenon in a bibliometric context. This research is based on empirical analysis, which may affect the generalizability of the research. A more in-depth technique, such as interviews or other qualitative measures, in the future may assist a more thorough investigation of the students’ coping strategies. Furthermore, if the university authority, administrative staffs and teachers could be selected as respondents, the study might produce more interesting findings. An epidemiologically robust survey on a specific group of people can discover the phenomenon further.

Despite some limitations, the study indicates numerous implications for society. First, the university authority should be aware of the students’ coping strategies. In particular, students who live without parents or relatives should be taken care of properly during the outbreak. Second, to help students cope with the mental pressure, university authorities may think about arranging or organizing programs such as an online experience-sharing competition, and encourage students by offering rewards or financial aids. Finally, required food and healthcare materials should be supplied to ensure the students’ safety.

The study sheds light on the coping strategies of Chinese students during the COVID-19 pandemic. Due to the lockdown policy, many students have had to stay in university hostels with no permission to go out. The coping strategy was as challenging for students at various universities in China for COVID-19 as for any other pandemic ^5^. The overall findings of the study clearly indicate a significant relationship between university students and their coping strategies during the COVID-19 in China. Increasing psychological pressure on students – consistent with our study finding that 48.3% of respondents believed that during the epidemic period everything would collapse – affect the students’ usual coping strategies and also their regular academic activities such as class attendance and assignments. As a result, ideal and safe coping strategies should be identified for the students in order to face any epidemic challenges in the future ^10^, which may help to ensure sustainable educational development in the world. Prioritizing research on mental health, anxiety and students’ coping strategies along with psychological effects is necessary.

## Data availability

### Underlying data

Figshare: COVID-19 and Coping Strategies of Anxiety in China, https://doi.org/10.6084/m9.figshare.12801395.v3 ^17^. 

Data are available under the terms of the Creative Commons Attribution 4.0 International license (CC-BY 4.0).

### Extended data

This project contains the following extended data:

- Respondent answers concerning their lives during the COVID-19 pandemic
- Survey sent to students
