# Supplementary material for: Coping strategies of students for anxiety during the COVID-19 pandemic in China: a cross-sectional study
Source: F1000Res. 2020 Sep 10;9:1115. [Version 1] doi: 10.12688/f1000research.25557.1 (PMC7682494; doi:10.12688/f1000research.25557.1)
Supplement: Peer review history

# Peer review: Reviewer response for version 1

**DOI:** 10.5256/f1000research.28205.r75079

**Published:** 2020-11-20

**Authors:** Dario Salerno

**Affiliations:** 1 Department of Management and Quantitative studies, University of Naples “Parthenope”, Naples, Italy

**Comment 1:**

 Using an amazing dataset, the authors examine the coping strategies of Chinese students during the COVID-19 pandemic in China. The analyzed sample consists of 559 responses to an online survey in which they captured information about students’ lives during the COVID-19 outbreak, actions to control anxiety, and what students care about during the pandemic. They show that coping strategies and survival techniques were necessary due to high levels of anxiety and psychological pressure during the COVID-19 pandemic. In addition, during the period considered, the authors argue that the students analyzed also had psychological problems such as lack of sleep, emotional support, mental support, and social appeal.

 The topic analyzed is of particular interest and extremely current, given the changes that students have had in their lives during the pandemic period.

 Additionally, the paper is well organized and provides some helpful insights into our understanding of how institutions can manage the negative effects of the pandemic crisis and what the consequences may be on very young people. It also opens very interesting ideas for future research. It also sheds light on the effectiveness of the measures taken during the pandemic by the institutions.

**Comment 2:**

 The authors make some reasonable theoretical insights into the introduction of the paper. In particular, they explain well theoretically the channel by which coping strategies and survival techniques are necessary during the pandemic period. Also, the motivation and contribution are clear and the structure/writing is mostly easy to follow.

**Comment 3:**

 The methodology is articulate in most of its parts. The results are in-depth analyzed and discussed. In their current form, they have a substantive discussion in relation to the relevant specialized literature.

**Comment 4:**

 ​​​​​The implications for research and study limitations are addressed and depict and integrate the original outputs of the study.

Is the work clearly and accurately presented and does it cite the current literature?

Yes

If applicable, is the statistical analysis and its interpretation appropriate?

Yes

Are all the source data underlying the results available to ensure full reproducibility?

Yes

Is the study design appropriate and is the work technically sound?

Yes

Are the conclusions drawn adequately supported by the results?

Yes

Are sufficient details of methods and analysis provided to allow replication by others?

Yes

Reviewer Expertise:

Banking, Financial Markets, Risk of Bankrupcty, Corporate Governance, CSR.

I confirm that I have read this submission and believe that I have an appropriate level of expertise to confirm that it is of an acceptable scientific standard.

# Peer review: Reviewer response for version 1

**DOI:** 10.5256/f1000research.28205.r71184

**Published:** 2020-10-07

**Authors:** Nizar Alsharari

**Affiliations:** 1 Business School, Higher Colleges of Technology, Sharjah, United Arab Emirates

The paper is still not sufficiently analyzed through the theoretical framework discussed in the early parts of the paper. Hence, I believe contribution to the literature is still limited.

 The paper has potential, but you need to refocus your literature review, provide more detail on the new ideas and analyze further your findings through your suggested theoretical framework. The literature should be updated as well....

 The discussion and results are not presented clearly and analyzed appropriately. The conclusions are not adequately linking together the other elements of the paper.

Is the work clearly and accurately presented and does it cite the current literature?

Partly

If applicable, is the statistical analysis and its interpretation appropriate?

Partly

Are all the source data underlying the results available to ensure full reproducibility?

Partly

Is the study design appropriate and is the work technically sound?

Partly

Are the conclusions drawn adequately supported by the results?

Partly

Are sufficient details of methods and analysis provided to allow replication by others?

Partly

Reviewer Expertise:

Accounting and Finance

I confirm that I have read this submission and believe that I have an appropriate level of expertise to state that I do not consider it to be of an acceptable scientific standard, for reasons outlined above.

# Peer review: Reviewer response for version 1

**DOI:** 10.5256/f1000research.28205.r71187

**Published:** 2020-09-23

**Authors:** Delia Deliu

**Affiliations:** 1 Faculty of Economics and Business Administration, West University of Timisoara, Timișoara, Romania

**Comment 1 – General comment:**

 The manuscript **“Coping strategies of students for anxiety during the COVID-19 pandemic in China: a cross-sectional study” **by **Mohammad Nurunnabi, Syed Far Abid Hossain Hossain, Karuthan Chinna, Sheela Sundarasen, Heba Bakr Khoshaim, Kamilah Kamaludin, Gul Mohammad Baloch, Areej Sukayt **and **Xu Shan **explores a theme of interest referring to the negative outcomes and consequences of remote studying (in general), respectively coping strategies of university students (in particular) in China, in the context of the new Coronavirus (Covid-19) pandemic.

 The topic approached by the authors is a hot topic these days and of major importance to all actors on the market.

 The paper has a very up-to-date approach, as tackling the Covid-19 negative impact is one of the most important concerns of worldwide economics. Moreover, having solutions for this time of crisis means having an efficient sustainable university management and a healthy governance, thus authors have approached this hot issue from the most pertinent point of view.

**Comment 2:**

 In ***section 1. Introduction***, you efficiently succeeded in creating reader interest in the topic and did well in hatching the broad foundation for the problem that leads to the study, the study being adequately placed within the larger context of the scholarly literature, as well as within the sensitive socio-economic context generated by the Covid-19 pandemic. Since you are working within a particular theoretical framework, the line of inquiry is introduced and discussed early, in the introduction, as well as in the literature review.

 The purpose of the research is clearly outlined in the Introduction and, therefore, provides a specific and accurate synopsis of the overall purpose of the study, since you briefly define and delimit the specific area of the research.

 The ***literature review*** provides the background and context for the research problem and is successful in establishing the need for the research and sharing the results of other studies that are closely related to the research.

 However, a more enhanced ***literature review*** (comprising more references) – are needed, in order to better place the study in the broad context and highlight why it is relevant, as well as to outline the factors to be taken into consideration in regards to psychological factors affecting individuals during endemics/pandemics/social crises.

 Therefore, in order to strengthen and improve this study, I suggest adding a more comprehensive literature review on the debated concepts.

**Comment 3:**

 Regarding the ***research materials and methods***, the authors have provided a well thought-out rationale for your decision to use the design, methodology, and analyses you have selected.

**Comment 4:**

***Section 3. Results*** clearly provide a concise and precise description of this research, as well as the experimental conclusions that can be drawn from the performed analysis. The authors clearly discuss the results and explain how they can be interpreted in perspective of initial research assumptions and previous studies.

**Comment 5:**

 The paper reached valuable ***conclusions*** regarding the negative implications of remote studying in the context of the new Coronavirus (Covid-19) pandemic, namely those induced by anxiety, also outlining strategies used by students in relation to coping with all the negative psychological effects.

**Comment 6 – Final comment:**

 In conclusion, the study, which is focused on a very relevant topic, presents a convincing structure and is written fluently, the research methodology being adequate and robust.

 Conversely, I consider the authors could improve it by adding more bibliographic references in order to outline the intercorrelation between the debated concepts.

Is the work clearly and accurately presented and does it cite the current literature?

Partly

If applicable, is the statistical analysis and its interpretation appropriate?

Yes

Are all the source data underlying the results available to ensure full reproducibility?

Yes

Is the study design appropriate and is the work technically sound?

Yes

Are the conclusions drawn adequately supported by the results?

Yes

Are sufficient details of methods and analysis provided to allow replication by others?

Yes

Reviewer Expertise:

accounting and financial audit; corporate governance, CSR and sustainability in times of crisis; financial auditor’s responsibilities and liabilities in a sensitive socio-economic context; quality of (non-)financial reporting and external auditing; the impact of Blockchain on the accounting and auditing profession; impact of technology integration in education; quality of education

I confirm that I have read this submission and believe that I have an appropriate level of expertise to confirm that it is of an acceptable scientific standard.

## References

[1] : Financial Audit vs. Corporate Governance Practices – A Viewpoint on Current Challenges and Perspectives in the Time of CoVid-19, Ovidius University Annals. *Economic Sciences Series*.2020;

[2] : The Intertwining between Corporate Governance and Knowledge Management in the Time of Covid-19 – A Framework, Journal of Emerging Trends in Marketing and Management 2020, 1(1), The Bucharest University of Economic Studies Publishing House, Emerging Trends in Marketing and Management (E.T.I.M.M.) International Conference.2020;

[3] : The Impact of a Socio-Economic Crisis on Corporate Governance Effectiveness and Sustainable Development. Acumen: The Current New Coronavirus (Covid-19) Pandemic, 35th I.B.I.M.A. (International Business Information Management Association) International Conference.2020;
